# Supplementary figures and images for: A Genome-Wide RNAi Screen in Caenorhabditis elegans Identifies the Nicotinic Acetylcholine Receptor Subunit ACR-7 as an Antipsychotic Drug Target
Source: PLoS Genet. 2013 Feb 28;9(2):e1003313. doi: 10.1371/journal.pgen.1003313 (PMC3585123; doi:10.1371/journal.pgen.1003313)

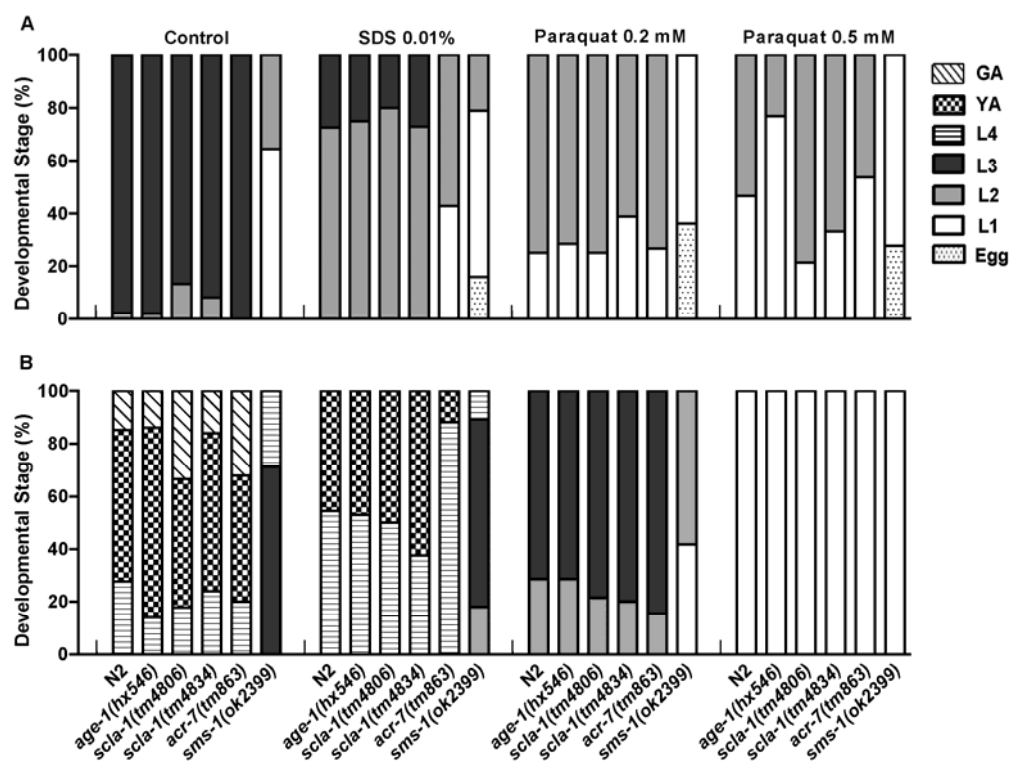

Supplement: Figure S1 — RNAi screen mutants did not suppress stressor-induced developmental delay. (A) Developmental delay seen with SDS or paraquat after 24 hours. (B) Developmental delay seen with SDS or paraquat after 48 hours. For control conditions, vehicle alone was applied to the agar. Paraquat at 0.5 mM produced significant lethality at 48 hours (data not shown). (PDF) [file pgen.1003313.s001.pdf]
