# Supplementary material for: A Genome-Wide RNAi Screen in Caenorhabditis elegans Identifies the Nicotinic Acetylcholine Receptor Subunit ACR-7 as an Antipsychotic Drug Target
Source: PLoS Genet. 2013 Feb 28;9(2):e1003313. doi: 10.1371/journal.pgen.1003313 (PMC3585123; doi:10.1371/journal.pgen.1003313)
Supplement: Table S1 — List of mutant strains used in these studies. Dr. L. Chen (University of Minnesota, Minneapolis, MN) provided LH202. Dr. C. Hunter (Harvard University, Cambridge, MA) provided NL4256. Dr. Y. Jin (UCSD, La Jolla, CA) provided CZ9957. Dr. S. Mitani (NBP-Japan) provided F22B3.8(tm4806) IV and F22B3.8(tm4534) IV. All other strains used in this work, except strains EAB1 through EAB211, were provided by the Caenorhabditis Genetics Center, which is funded by the NIH National Center for Research Resources (NCRR). NA, not available. (PDF) [file pgen.1003313.s002.pdf]

Table S1. Strains

Strain Name	Genotype
CB113	*unc-17(e113) IV*
CB211	*lev-1(e211) IV*
CB933	*unc-17(e245) IV*
CB1072	*unc-29(e1072) I*
CE1255	*cep-1(ep347) I*
CZ9957	*gtl-2(n2618) IV*
DA453	*eat-2(ad453)II*
DA465	*eat-2(ad465)II*
DA1674	*acr-19(ad1674) I*
EAB1	*sms-1(ok2399) IV*
EAB21	N2 *mchEx21* [*Pacr-7::GFP, rol-6(su1006)*]
EAB22	N2 *mchEx22* [*Pacr-7::GFP, rol-6(su1006)*]
EAB23	N2 *mchEx23* [*Pacr-7::GFP, rol-6(su1006)*]
EAB24	N2 *mchEx24* [*Pacr-7::GFP, rol-6(su1006)*]
EAB39	*acr-7(tm863) II mchEx39* [*Pacr-7::acr-7::GFP, Pmyo-3::DsRed2*]
EAB40	*acr-7(tm863) II mchEx40* [*Pacr-7::acr-7::GFP, Pmyo-3::DsRed2*]
EAB41	*acr-7(tm863) II mchEx41* [*Pacr-7::acr-7::GFP, Pmyo-3::DsRed2*]
EAB100	*age-1(hx546) II*
EAB101	*scla-1(tm4806) IV*
EAB102	*scla-1(tm4534) IV*
EAB103	*ins-22(ok3616) III*
EAB200	*acr-7(tm863) II*
EAB201	*acr-16(ok789) V*
EAB207	*acr-7(tm863) II mchEx207* [*Pmyo-2::acr-7, Pmyo-3::DsRed2*]
EAB208	*acr-7(tm863) II mchEx208* [*Pmyo-2::acr-7, Pmyo-3::DsRed2*]
EAB211	*acr-7(tm863) II mchEx211* [*Pmyo-2::acr-7::GFP, Pmyo-3::DsRed2*]
FX863	*acr-7(tm863) II*
LH202	*gtl-2(tm1463) IV*
MT6205	*cha-1(n2411) IV*
NA	*F22B3.8(tm4806) IV*
NA	*F22B3.8(tm4534) IV*
NC467	*acr-5(ok205) III*
NG39	*ina-1(gm39) III*
NL4256	*rrf-3(pk1426) II*
PR1152	*cha-1(p1152) IV*
RB918	*acr-16(ok789) V*
RB1132	*acr-14(ok1155) II*
RB1142	*lron-6(ok1119) I*
RB1172	*acr-15(ok1214) V*
RB1195	*acr-8(ok1240) X*
RB1226	*acr-18(ok1285) V*

Table S1. Strains	
RB1250	*acr-21(ok1314) III*
RB1263	*acr-11(ok1345) I*
RB1559	*acr-2(ok1887) X*
RB1659	*acr-3(ok2049) X*
RB1854	*sms-1(ok2399) IV*
RB2119	*acr-23(ok2804) V*
RB2250	*puf-6(ok3044) II*
RB2294	*acr-6(ok3117) I*
RB2295	*acr-10(ok3118) X*
RB2594	*ins-22(ok3616) III*
TG12	*cep-1(lg12501) I*; *unc-119(ed4) III*; *gtIs1* [*CEP-1::GFP*, *unc-119(+)*]
TJ1	*cep-1(gk138) I*
TJ1052	*age-1(hx546) II*
VC188	*acr-12(ok367) X*
VC649	*acr-9(ok933) X*
VC731	*unc-63(ok1075) I*
VC1408	*magi-1(gk657) IV*
XY1054	*cep-1(lg12501) I*
ZZ15	*lev-8(x15) X*
