# Supplementary material for: A Genome-Wide RNAi Screen in Caenorhabditis elegans Identifies the Nicotinic Acetylcholine Receptor Subunit ACR-7 as an Antipsychotic Drug Target
Source: PLoS Genet. 2013 Feb 28;9(2):e1003313. doi: 10.1371/journal.pgen.1003313 (PMC3585123; doi:10.1371/journal.pgen.1003313)
Supplement: Table S2 — List of primers used for plasmid constructions. (PDF) [file pgen.1003313.s003.pdf]

Table S2. Primers	
Pacr-7::gfp primers	
Primer Name	Sequence
ACR1	5'-AAAACTGCAGTTGAGCAAGTGTGGCTGTTC-3'
ACR2	5'-TCCCCCCGGGTAATTTTCAAAATTAAAATTGTGGAA-3'
Pacr-7::acr-7::gfp primers	
Primer Name	Sequence
ACR3	5'-TTTCTGCAGTTGAGCAAGTGTGGCTGTTC-3'
ACR4	5'-TTTGGTACCCATCCAATATTTTGATAGGTGGAA -3'
Pmyo-2::acr-7 primers	
Primer Name	Sequence
ACR5	5'-TTTTGCTAGCATGATGGTTCAATCAATTCAAAT-3'
ACR6	5'TTTTGGTACCTCAATCCAATATTTTGATAGGTG-3'
Pmyo-2::acr-7::gfp primers	
Primer Name	Sequence
ACR7	5'-TTTTGTCGACATGATGGTTCAATCAATTCAA-3'
ACR8	5'-TTTTCGGCCGAATCCAATATTTTGATAGGTG-3'
